# Retraction: Moxibustion Modulates Sympathoexcitatory Cardiovascular Reflex Responses Through Paraventricular Nucleus

**DOI:** 10.3389/fnins.2020.630045

**Published:** 2021-02-08

**Authors:** Frontiers Editorial Office

The journal retracts the 21 January 2019 article cited above.

Following publication, concerns were raised regarding the ethical approval of the moxibustion procedure used in the study. After an investigation by the Editorial Office it was confirmed that the moxibustion procedure was not reviewed nor approved by the University of California, Irvine's Institutional Animal Care and Use Committee.

The authors concur with the retraction and sincerely regret any inconvenience this may have caused to the reviewers, editors, and readers of Frontiers in Neuroscience.

